# Hepatocyte Growth Factor, a Determinant of Airspace Homeostasis in the Murine Lung

**DOI:** 10.1371/journal.pgen.1003228

**Published:** 2013-02-14

**Authors:** Carla Calvi, Megan Podowski, Armando Lopez-Mercado, Shana Metzger, Kaori Misono, Alla Malinina, Dustin Dikeman, Hataya Poonyagariyon, Leslie Ynalvez, Roshanak Derakhshandeh, Anne Le, Mark Merchant, Ralph Schwall, Enid R. Neptune

**Affiliations:** 1 Division of Pulmonary and Critical Care Medicine, Johns Hopkins University School of Medicine, Baltimore, Maryland, United States of America; 2 Department of Pathology, Johns Hopkins University School of Medicine, Baltimore, Maryland, United States of America; 3 Department of Translational Oncology, Genentech, South San Francisco, California, United States of America; University of Pittsburgh, United States of America

## Abstract

The alveolar compartment, the fundamental gas exchange unit in the lung, is critical for tissue oxygenation and viability. We explored hepatocyte growth factor (HGF), a pleiotrophic cytokine that promotes epithelial proliferation, morphogenesis, migration, and resistance to apoptosis, as a candidate mediator of alveolar formation and regeneration. Mice deficient in the expression of the HGF receptor *Met* in lung epithelial cells demonstrated impaired airspace formation marked by a reduction in alveolar epithelial cell abundance and survival, truncation of the pulmonary vascular bed, and enhanced oxidative stress. Administration of recombinant HGF to tight-skin mice, an established genetic emphysema model, attenuated airspace enlargement and reduced oxidative stress. Repair in the TSK/+ mouse was punctuated by enhanced akt and stat3 activation. HGF treatment of an alveolar epithelial cell line not only induced proliferation and scattering of the cells but also conferred protection against staurosporine-induced apoptosis, properties critical for alveolar septation. HGF promoted cell survival was attenuated by akt inhibition. Primary alveolar epithelial cells treated with HGF showed improved survival and enhanced antioxidant production. In conclusion, using both loss-of-function and gain-of-function maneuvers, we show that HGF signaling is necessary for alveolar homeostasis in the developing lung and that augmentation of HGF signaling can improve airspace morphology in murine emphysema. Our studies converge on prosurvival signaling and antioxidant protection as critical pathways in HGF–mediated airspace maintenance or repair. These findings support the exploration of HGF signaling enhancement for diseases of the airspace.

## Author Summary

The airspace compartment of the mammalian lung, comprised of spherical sacs termed alveoli, harbors the architecture, cellular composition, and molecular armamentarium to perform the critical function of gas exchange or oxygen uptake. Despite the necessity of this alveolar compartment for organismal viability, the mechanism by which alveoli are formed and maintained is obscure. Furthermore, no treatments are currently available that can regenerate the airspace once damaged. In this manuscript, we sought to determine whether hepatocyte growth factor, a cytokine with a functional armamentarium that subserves the critical events of alveolar formation (epithelial proliferation, migration, resistance from apoptosis and angiogenesis), could be an important mediator of alveolar formation and airspace maintenance. Our simple paradigm was that critical homeostatic pathways for the lung should operate both in lung formation and in lung maintenance/regeneration. Using an informative battery of mouse models and cell lines, we show that hepatocyte growth factor is a determinant of alveolar formation and that the enhancement of hepatocyte growth factor signaling can both protect and repair the airspace from pathologic airspace enlargement or emphysema.

## Introduction

One approach to identifying mediators of alveolar formation and regeneration in the mammalian lung is to delineate the elemental events that attend airspace formation and then systematically investigate candidate proteins that harbor a compatible signaling repertoire in animal or cellular model systems. From a developmental perspective, the eruption of alveolar septae from a primordial saccule in early postnatal murine life requires localized epithelial proliferation, migration and resistance to apoptosis [1], [2]. Furthermore, epithelial morphogenesis must be accompanied by a microvasculature that permits efficient diffusion of gases from the airspace lumen to the systemic vascular bed. A candidate mediator of such events is hepatocyte growth factor (HGF). HGF is a pleiotrophic cytokine that promotes epithelial proliferation, morphogenesis, migration and survival [3], [4]. HGF is also known to induce angiogenesis and inhibit epithelial apoptosis. We sought to determine whether HGF is critical for alveolar formation and might have a therapeutic role in alveolar regeneration.

The HGF/c-Met signaling pathway incorporates all of the features of a key alveolar survival factor [5]. HGF is expressed with its receptor (c-Met or *Met*) in the vertebrate lung parenchyma. Upon HGF binding, c-Met undergoes autophosphorylation which initiates the recruitment of a variety of downstream signal transduction molecules (reviewed in [6]). In selective models, HGF signaling supports postpneumonectomy lung growth, induces branching morphogenesis and ameliorates inflammatory lung injury [7]–[9]. Unfortunately, these artificial models imperfectly approximate the physiologic events required for alveolar formation and maintenance. Moreover, since elevated local and systemic HGF levels in patients with lung injury correlate with disease severity and poor outcomes, whether HGF is a participant in airspace repair or a marker of ongoing injury is a subject of controversy [3]. When HGF is administered during neonatal hyperoxia or early in the course of bleomycin lung injury, some measure of protection against lung damage is observed. However, no studies have addressed whether HGF/c-Met signaling is 1) required for alveolar formation or 2) has therapeutic value in animals with established pathologic airspace enlargement. Our studies utilize both loss-of-function and gain-of-function strategies in the murine lung to investigate the function of this pathway in murine airspace formation and airspace repair. To delineate a developmental role, we present a postnatal interrogation of mice deficient in HGF signaling in the airspace epithelia. To investigate mechanisms of airspace repair, we use the TSK/+ mouse model of genetic emphysema, a convenient platform to evaluate strategies for airspace regeneration. In studies here, we both assess whether HGF infusion can improve airspace caliber in this model and evaluate downstream pathways engaged in the therapeutic response. The goal is to invoke candidate mechanisms for HGF-mediated airspace repair with possible broad therapeutic utility.

In the present study, we find that mice deficient in *Met* expression in alveolar epithelial cells exhibit impaired airspace morphology accompanied by a reduced abundance and survival of alveolar type II cells. We also show a reduction of vascularization in the lung parenchyma of *Met*-deficient mice, suggesting an intimate morphogenic connection between the epithelial and endothelial compartments. Pharmacologic augmentation of HGF signaling in a murine model of emphysema reverses airspace enlargement in the adult lung and is marked by akt and stat3 activation. Finally, in whole cell assays using primary and immortalized alveolar epithelial cells, we establish that hepatocyte growth factor signaling promotes cell survival, induces proliferation and scattering of alveolar epithelial cells, confers protection against cell death via akt activation and mediates antioxidant production. These data 1) support a critical role for HGF signaling in alveolar development and regeneration and 2) implicate downstream prosurvival signaling as a contributor to the airspace maintenance and reparative effects of HGF. Importantly, the studies suggest that developmental strategies for airspace formation are recapitulated in reparative contexts.

## Results

### Characterization of HGF and c-Met expression in the murine airspace

**Figure 1:**
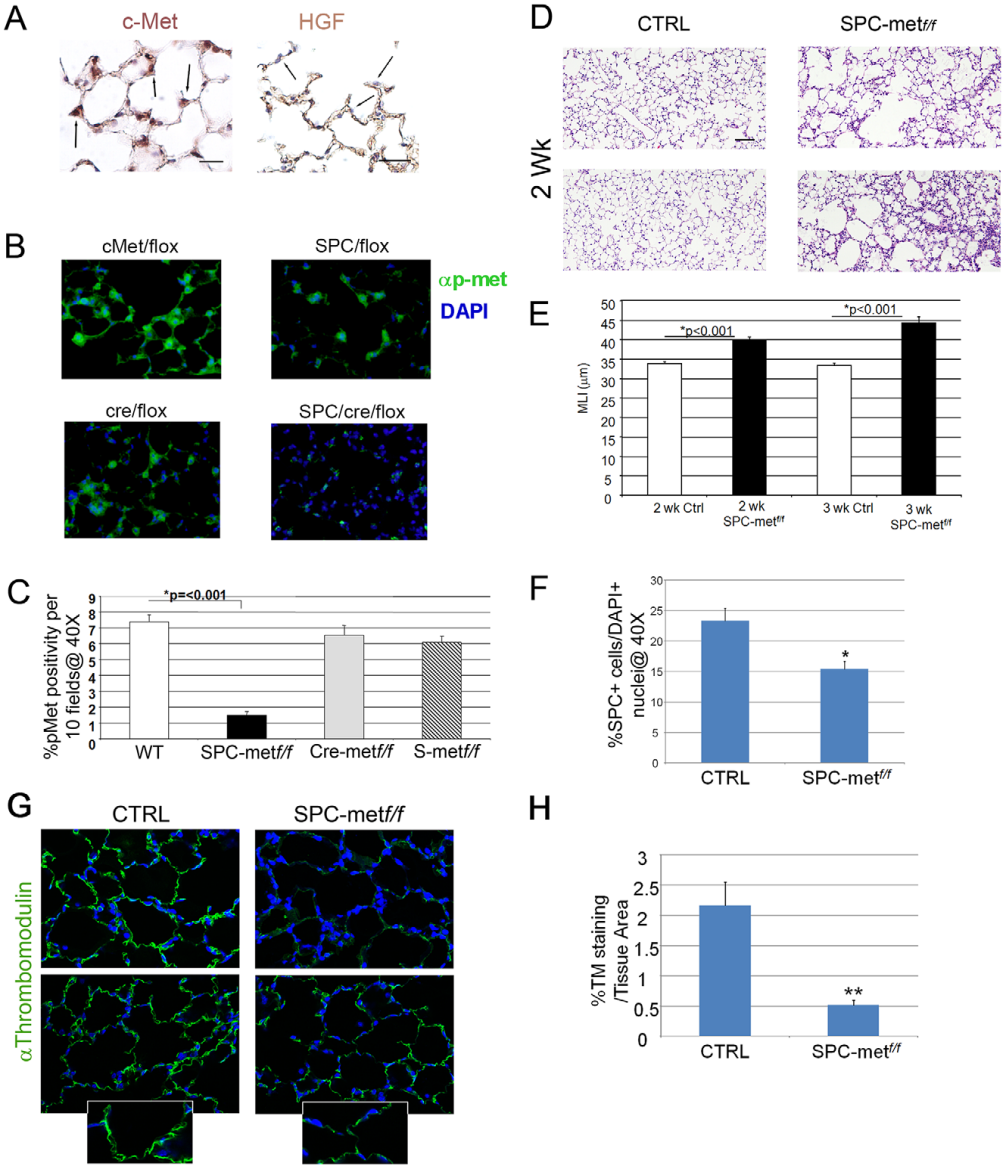
Generation and characterization of mice deficient in *Met* expression in alveolar epithelial cells. A. Right panel-Representative immunohistochemical staining of c-Met in 2 week old mouse lungs. 40× magnification. N = 4–6 mice. Arrows denote expression in type II epithelial cells. Left panel-Representative immunohistochemical staining for HGF in 2 week old mouse lungs. 40× magnification. N = 4–6 mice. Scale bar (L): 25 µm, (R): 50 µm. Arrows denote exclusion of HGF from alveolar epithelial cells with apparent localization to the interstitium. B. Representative fluorescent immunohistochemistry of phosphorylated c-Met in mice deficient in *Met* and bitransgenic controls. Green-p-Met. 40× magnification. N = 4–6 mice. Scale bar: 25 µm. C. Quantitative immunohistochemistry of p-met expression in the airspace of *Met*-deleted mice and controls. D. Representative histology of mice deficient in airspace *Met* expression and controls at two weeks of age. Note patchy airspace enlargement in the targeted mice. Scale bar: 100 µm. E. Airspace dimension by morphometry in *Met*-deficient mice and controls at 2 and 3 weeks of age. F. Quantitation of SPC+ cells in the airspace by SPC immunohistochemistry in *Met*-deficient mice compared with control bitransgenic mice. *p<0.05. G. Representative thrombomodulin immunohistochemical staining of the microvascular bed in the lung parenchyma of Met deficient mice compared with controls. Inset shows reduced staining in the alveolar epithelial walls. 40× magnification, inset 100×. N = 5–7 mice per genotype. H. Quantitative immunohistochemistry of thromobomodulin staining of *Met*-deficient mice and controls. **p<0.01.

In order to invoke a role of the HGF/c-Met pathway in alveolar morphogenesis, we first established that the ligand and receptor are expressed in alveoli. Using immunohistochemistry, we show that c-Met is expressed in alveolar type II cells in the lungs of two- week old C57Bl/6 mice (Figure 1A). We also show that the HGF ligand is expressed diffusely in the interstitium of the alveolar septum. HGF is notably excluded from alveolar epithelial cells, consistent with its known localization and deposition in other tissues (Figure 1A and Figure S1A). Coimmunostaining for an alveolar epithelial marker (SPC) and c-Met in 2 week old lungs shows that the sites of c-Met expression are alveolar epithelial cells, airway epithelial cells and a subset of alveolar macrophages (Figure S1B).

### Deletion of *Met* in alveolar epithelial cells impairs airspace morphogenesis, alveolar epithelial survival, lung microvascular development, and prosurvival signaling

Having established that HGF and c-Met are expressed in the developing murine lung, we investigated whether this pathway was critical for alveolarization. Yamamoto recently showed that mice with an alveolar epithelial cell specific deletion of *Met* had impaired late embryonic lung development, implicating the HGF/c-Met pathway in late lung development [10]. However, a dedicated analysis of the postnatal phenotype was not pursued. We generated mice deficient in *Met* expression in alveolar epithelial type II cells (AECII). Conditional alveolar epithelial cell specific deletion of *Met* was achieved by crossing the well characterized SPC-rtta;otet-Cre cassette provided by Dr. Jeffrey Whitsett into *Met^f/f^* mice that harbor an inactivating conditional deletion in exon 16 provided by Dr. Snorri Thorgeirsson [11], [12]. These tritransgenic mice (*SPC-rtta/+;otet-Cre;Met^f/f^*, termed *SPCMet^f/f^*) were treated with doxycycline from conception and harvested at two and three weeks of age. The doxycycline-treated tritransgenic mice were normal in birthweight and showed no gross extrapulmonary phenotypic or histologic abnormalities observed at 6 months. We examined activated c-Met (phosphorylated c-Met) by immunohistochemical staining in the airspace of doxycycline treated *SPCMet^f/f^* mice compared with bitransgenic or single transgenic controls. Alveolar epithelial staining for p-met was largely ablated in the *SPCMet^f/f^* mice, consistent with inducible compartmental deletion of *Met* (Figure 1B, 1C). Modest and marked increases in airspace caliber were seen in two and three week old doxy-treated *SPCMet^f/f^* mice, respectively, (Figure 1D, 1E). This finding suggested that c-Met expression in alveolar epithelial cells contributes to normal alveolarization.

Since HGF is a known epithelial mitogen and survival factor, we investigated whether AECII abundance was altered in the *SPCMet^f/f^* lung. By immunohistochemistry, we found reduced number of AECII cells in the 1 month old *SPCMet^f/f^* lung (Figure 1F and Figure S2). As alveolar epithelial and endothelial morphogenesis are often interdependent, we examined microvascular abundance in the mutant lung. By thrombomodulin staining and quantitative immunohistochemistry, we found a marked truncation in the pulmonary vascular bed of the mutant mice (Figure 1G, 1H). These data suggest that the architectural defects observed in the mutant mice are likely secondary to both the primary impairment in alveolar epithelial cell survival and secondary cell-nonautonomous effects on the microvascular bed.

**Figure 2:**
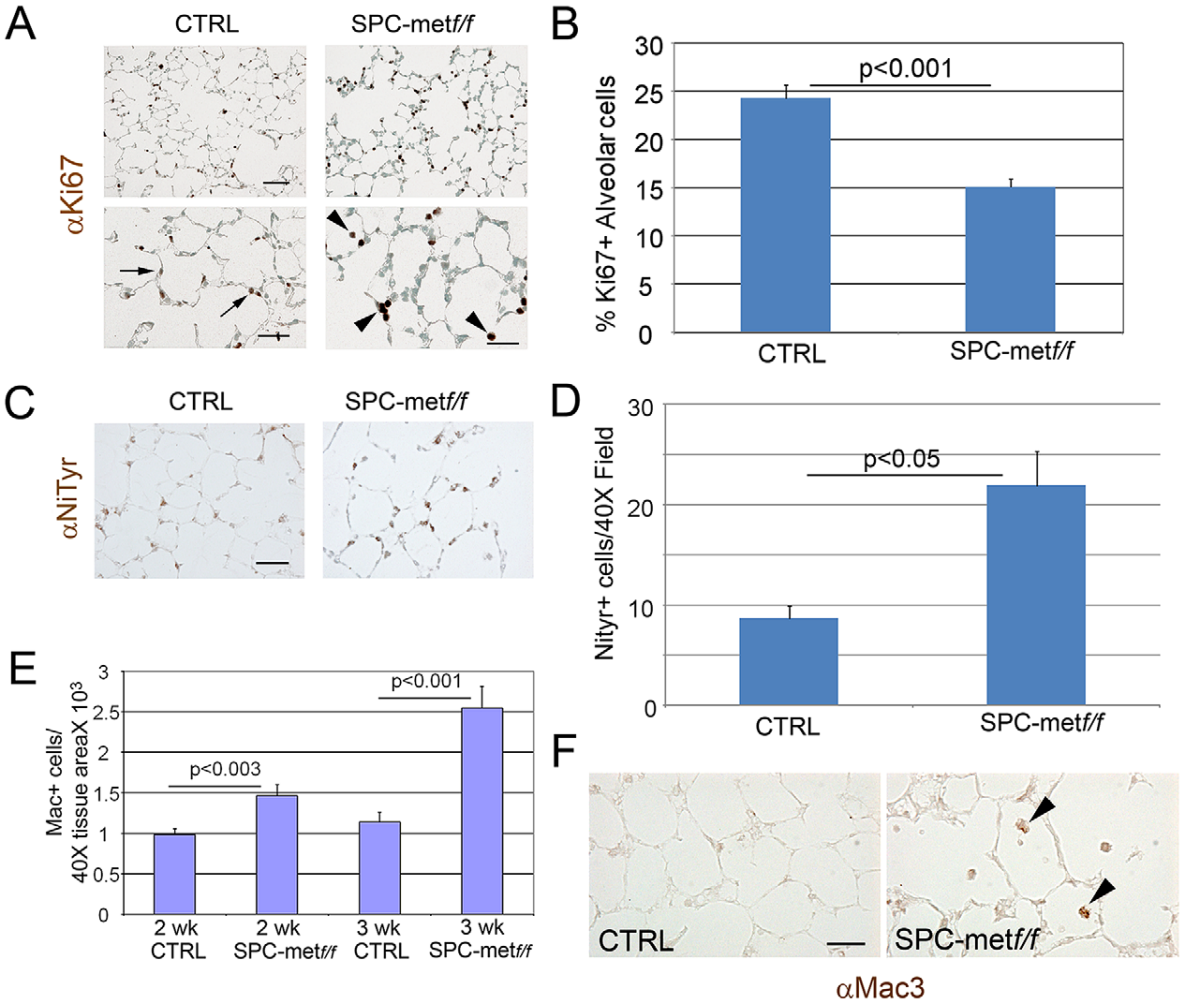
Increased oxidative stress, reduced alveolar cell proliferation, and increased inflammation in lungs of *Met*–deficient mice. A. Representative immunohistochemical staining for proliferation marker Ki67 in *Met*-deficient mice compared with controls. Arrows show increased alveolar cell proliferation in control lungs compared with mutant lungs. Arrowheads denote increased proliferation signal in alveolar macrophages in mutant lungs. Scale bar: 50 µm (top panel), 25 µm (bottom panel). B. Quantitative immunohistochemistry of Ki67 staining in the alveolar epithelium of *Met*-deficient mice and controls. C. Representative immunohistochemical staining (brown) for nitrotyrosine (NiTyr) in lungs of control and *SPCMet* ^f/f^ mice. Arrowheads denote positive staining. 20× magnification. N = 4 mice per genotype. Scale bar: 50 µm. D. Quantitative immunohistochemistry of nitrotyrosine staining in *SPCMet^f/f^* mice compared with controls. E. Macrophage abundance per Mac3 immunohistochemistry in *Met*-deficient lungs and controls at 2 and 3 weeks of age. Note increasing macrophage influx in mutant lungs. N = 4–6 mice per genotype. F. Representative immunohistochemical staining (brown) for macrophages (Mac3) in lungs of control and *SPCMet ^f/f^* mice at 3 weeks of age. Arrowheads denote positive staining. 40× magnification. N = 4–6 mice per genotype. Scale bar: 25 µm.

To further parse the alveolar epithelial cell phenotype of *Met*-deficient mice, we assessed measures of cell survival and stress in the alveolar compartment. The distribution of airspace proliferation, as assessed by Ki67 immunostaining, in the *SPCMet^f/f^* mice compared to wild type controls was different (Figure 2A). Whereas Ki67 staining was predominantly localized to airspace epithelial cells in the wild-type lung, staining was most prominent in the alveolar macrophages of the mutant lung (Figure 2A, 2B). By contrast, TUNEL staining, reflecting parenchymal cell death, was not enhanced in the mutant lung (data not shown). Of note, this combination of reduced target cell proliferation without enhanced cell death was also observed in hepatocyte-specific *Met* deletion [13]. Because oxidative stress in the airspace compartment can reduce proliferation and increase airspace dimension, we examined nitrotyrosine staining in the lungs of *SPCMet^f/f^* mice compared to wild type controls. We found a more than 50% increase in nitrotyrosine staining in the mutant lungs (Figure 2C, 2D). We assessed the expression of a panel of antioxidants in the lungs of mutant mice and found no significant change in the levels of NAD(P)H: quinone oxidoreductase-1 (*Nqo1*), heme oxygenase 1 (*Hmox1*) and glutamate-cysteine ligase catalytic subunit (*Gclc*) but a trend towards reduction in mutant mice (Revised Figure S3). Oxidant injury often associates with inflammation, especially macrophage influx, in various models of parenchymal lung disease. We indeed found increased macrophage abundance and proliferation in the lungs of *SPCMet^f/f^* mice (Figure 2E, 2F). Thus, the loss of c-Met expression in lung epithelial cells culminates in enhanced oxidative stress, reduced epithelial cell proliferation, mononuclear inflammation in the airspace compartment and a truncated microvascular bed. These insults likely confer the reduced AECII abundance and increased airspace dimension which define the airspace phenotype.

Loss of alveolar c-Met expression does not affect extracellular matrix expression or abundance. Since airspace homeostasis requires extracellular matrix integrity and HGF is known to attenuate fibrosis in animal models, we assessed the deposition of elastin and collagen in the lungs of wild-type and *Met*-deficient mice. Trichrome and modified Hart's staining showed no altered deposition of collagen or elastin respectively in the lungs of mutant mice compared with age-matched controls (Figure S4A, S4B).

### Enhanced HGF signaling increases proliferation and cell scattering but attenuates apoptosis in lung epithelial cells

**Figure 3:**
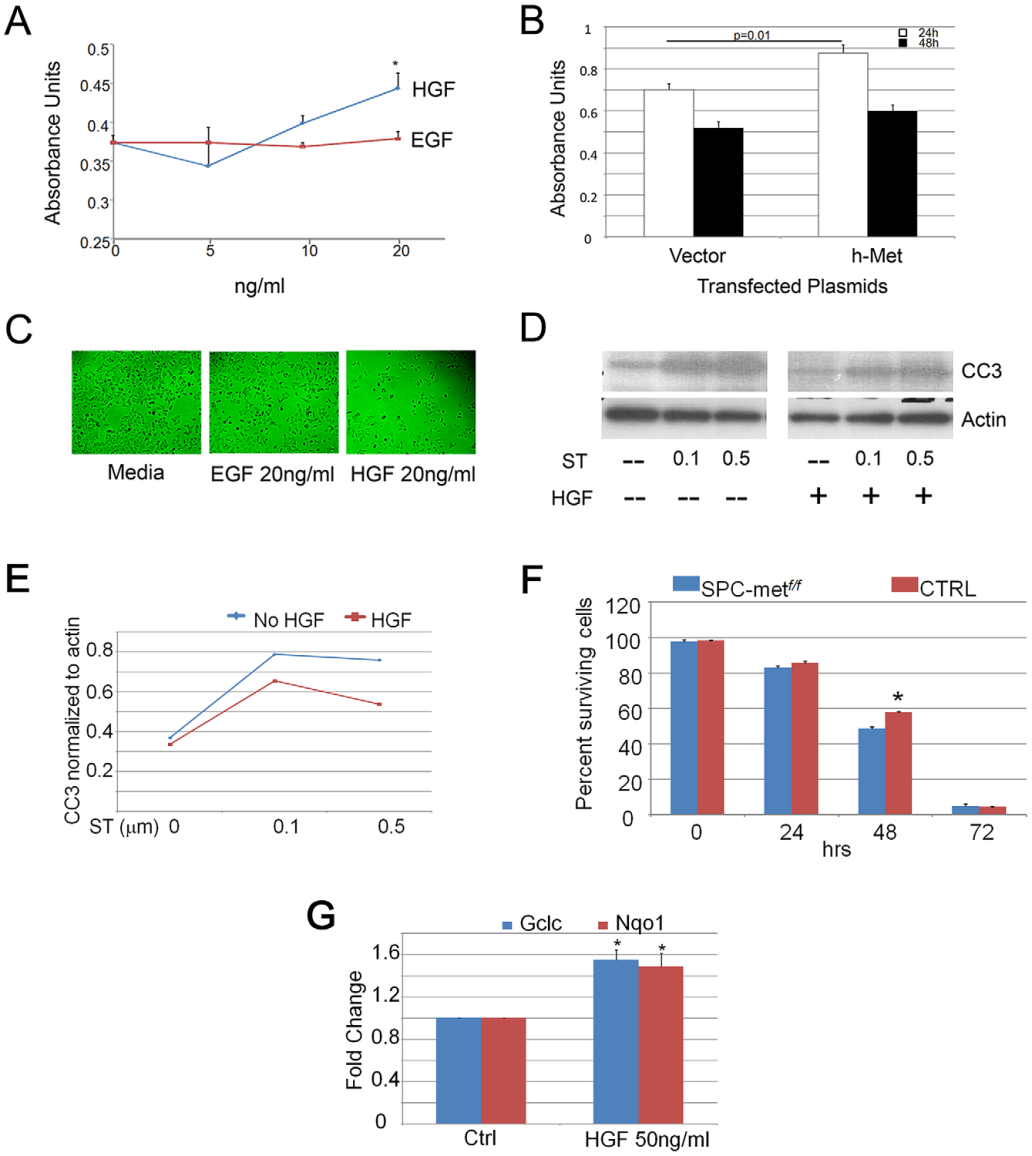
HGF signaling induces proliferation and scattering of MLE12 cells. A. Proliferation dose response of HGF and EGF treatment of MLE12 cells demonstrating a significant induction of proliferation by HGF. *p<0.05. B. Proliferation response of MLE12 cells transfected with *MET* or a control vector showing increased proliferation resulting from *MET* transfection. C. Cell dispersion images of MLE12 cells treated with EGF or HGF compared to media control. D. Effect of HGF treatment on staurosporine induced caspase 3 cleavage. ST-staurosporine. All cell experiments performed in triplicate. CC3-cleaved caspase 3. E. Densitometric quantitation of effect of HGF treatment on staurosporine induced caspase 3 cleavage. F. Survival time-course of primary alveolar epithelial cells from control and *Met*-deficient mice. *p<0.05. G. Relative expression of *Gclc* and *Nqo1* with HGF treatment of primary murine alveolar epithelial cells. *p<0.05 compared with control conditions.

To establish whether HGF exerts a direct effect on epithelial activities involved in alveolar septation, we employed pharmacologic and genetic enhancement of HGF signaling in MLE12 cells, an established murine alveolar epithelial cell line. Treatment of cells with recombinant HGF induced proliferation which was more robust at higher concentrations than that seen with EGF treatment, a known epithelial mitogen (Figure 3A). Similarly, transient transfection of human *MET* into MLE12 cells induced a significant increase in cell proliferation at 24 h when compared with vector control (Figure 3B). Because epithelial cell scattering approximates the cellular migratory activity that is required for alveolar formation and can be mediated selectively by HGF [14], [15], we determined whether HGF could promote such behavior in MLE12 cells. Treatment of serum-starved MLE12 with recombinant HGF resulted in marked dispersion compared with media or EGF controls (Figure 3C). Of note, the total cell counts were comparable in the EGF and HGF treated cells. Hepatocyte growth factor treatment also attenuated staurosporine-induced apoptosis in MLE12 cells (Figure 3D, 3E). In order to determine whether HGF enhances cell survival of primary alveolar epithelial cells, we performed a survival analysis of isolated murine alveolar type 2 (ATII) cells from wild-type and *SPCMet* ^f/f^ mice. Others have shown that HGF induces proliferation of primary rat alveolar type II cells [16], [17]. We found a significant increase in the survival of wild-type AEC cells at 48 h, consistent with a prosurvival effect of HGF signaling (Figure 3F). Using primary cells, we examined whether antioxidant and antiapoptotic signaling contributed to the short-term prosurvival effect of HGF. We found a significant induction of the antioxidants *Nqo1* and *Gclc* but no change in the expression of the antiapoptotic genes *Bcl2* and *Bax* with HGF treatment (Figure 3G and data not shown). This battery of in vivo and whole cell studies suggested that HGF/c-Met signaling utilizes both antioxidant and antiapoptotic effects to mediate selected components of the complex series of cellular events that are needed for alveolar formation and alveolar epithelial cell survival.

### TSK/+ mice, model of genetic emphysema, have altered HGF and Met expression

Given the multiple alveolar epithelial responses conferred by HGF signaling, we queried whether augmented HGF signaling might induce a protective or reparative response in murine models of emphysema. TSK/+ mice, a spontaneously mutant strain heterozygous for a mutant allele of the matrix protein fibrillin-1 which compromises fibrillin-1 activity, are a well-accepted model of genetic emphysema. They display alveolar septation defects that evolve into overt emphysema [18]. We recently showed that the TSK/+ airspace phenotype is partially attributable to matrix-associated susceptibility to oxidative stress resulting in alveolar cell death [19]. Before proceeding with an HGF augmentation strategy, we determined whether there are alterations in HGF expression and signaling in the TSK/+ mouse model of impaired septation. Real-time PCR, ELISA analysis and immunoblotting of whole lung specimens showed no alteration of HGF and c-Met expression in the PD14 TSK/+ lung compared with age-matched controls (Table S1, Figure S5A, S5B). However, at 2 months of age, activated HGF levels were reduced in the TSK/+ lung. By immunohistochemical analysis, although we saw some regions of reduced c-Met expression there was overall no consistent reduction in c-Met expression in the PD14 TSK/+ lung (Figure S5C, top). By contrast, we observed reduced and discontinuous expression of HGF within the interstitium of the airspace compartment of TSK/+ mice (Figure S5D, bottom). Given the antifibrotic effects of HGF in rodent models and the proposed role of alveolar myofibroblasts in alveolar homeostasis, we used alpha smooth muscle actin immunohistochemistry to gauge the abundance of alveolar myofibroblasts in the TSK/+ lung. We found few myofibroblasts in the alveolar compartment of both wild-type and TSK/+ mice as well as preserved abundance of SMCs in the airway submucosa (Figure S5E). These data suggest that airspace disorders characterized by abnormal extracellular matrix composition (e.g. TSK/+ mice) may exhibit altered HGF activation and deposition and that the TSK/+ mouse is an excellent model system to examine the therapeutic effects of HGF augmentation.

### Short-term augmentation of HGF signaling improves airspace enlargement and oxidative stress injury in TSK/+ mice

**Figure 4:**
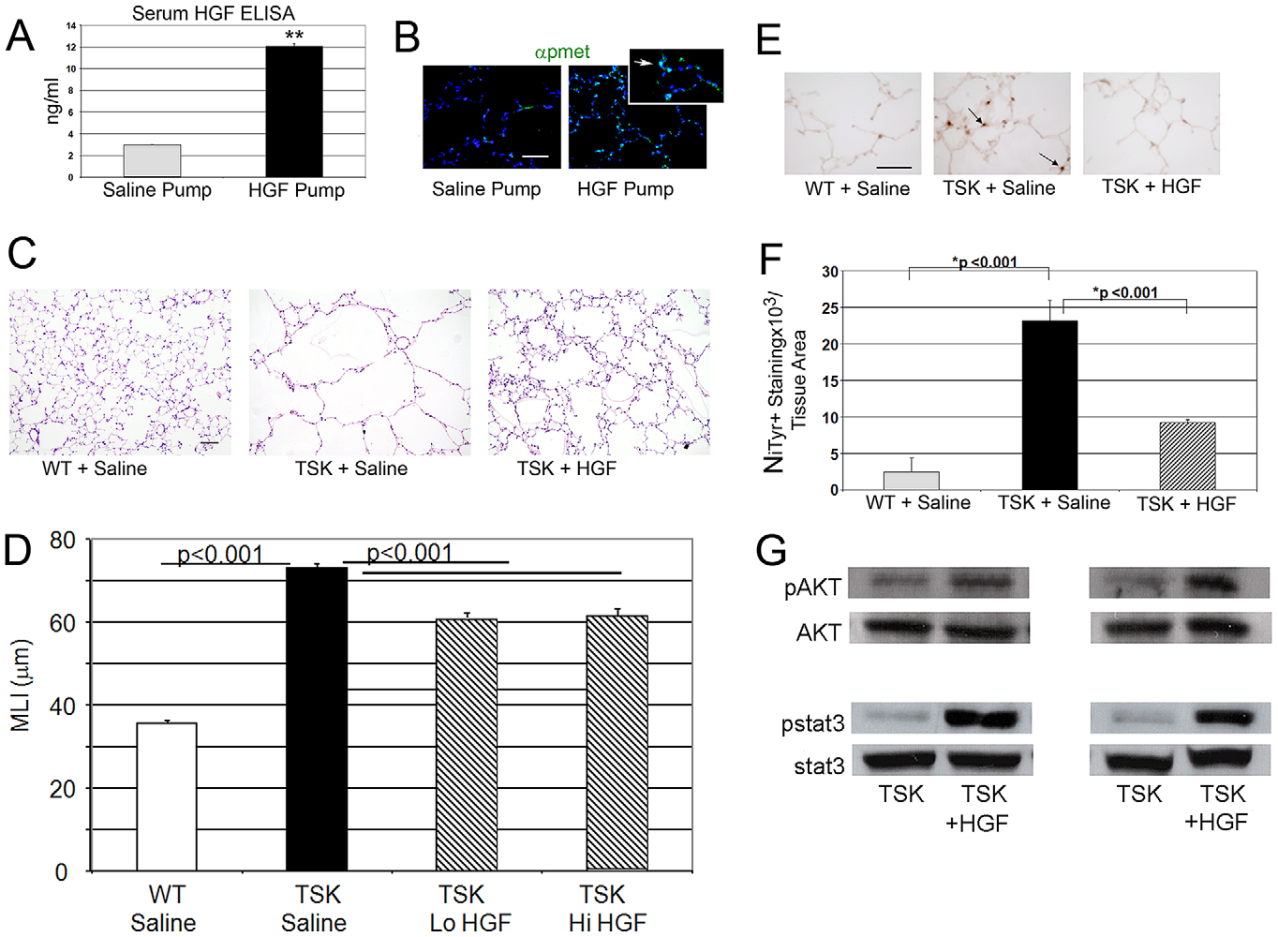
HGF treatment improves airspace caliber in TSK/+ mice. A. Serum HGF levels in mice treated with HGF infusion pumps at 50 µg/d. **p<.001 B. Phospho-Met immunofluorescent staining in lungs of mice treated with HGF infusion compared with PBS carrier infusion. Arrow in inset denotes phosphorylated c-Met (p-Met, green) in alveolar epithelial cells of HGF treated mice. Scale bar: 50 µm. C. Histology of TSK/+ lung treated with HGF compared with untreated controls. 20× magnification. Scale bar 100 µm. D. Morphometric assessment of airspace dimension in mice treated with HGF for 2 weeks compared with wild-type mice and untreated controls. Lo HGF-50 µg/d. Hi HGF-100 µg/d. E. Representative staining for oxidative stress marker nitrotyrosine in lungs of TSK/+ mice treated with HGF and controls. Arrow denotes staining in alveolar epithelial cells. 40× magnification. Scale bar 50 µm. F. Quantitative immunohistochemistry of nitrotyrosine staining in TSK/+ lungs treated with saline or HGF by infusion pump compared to wild-type controls. G. Representative immunoblotting of normalized phosphomediator levels (akt and stat3) in two TSK/+ mice treated with HGF compared with saline treated mice. N = 4–8 mice per group and treatment.

Since alveolar cell specific deletion of *Met* compromises alveolar formation, we examined whether enhanced HGF signaling might rescue the airspace phenotype in TSK/+ mice. A subcutaneous pump containing active, recombinant human HGF, kindly provided by Drs. Ralph Schwall and Mark Merchant at Genentech, or carrier protein was inserted into adult TSK/+ mice and wild type controls. The HGF pumps delivered 50 µg/day over a two week period. We measured human HGF levels in HGF pump mice by ELISA assay, comparing intratracheal and subcutaneous pump delivery of comparable doses (50 µg/d for 3 d). A marked elevation in serum HGF was observed after pump delivery but not intratracheal delivery (Figure 4A). Immunohistochemical staining for enhanced HGF signaling in the lung as evidenced by activated c-Met (p-Met) expression showed increased staining in the airspace compartment in the HGF-treated mice (Figure 4B). We assessed airspace morphology as an index of airspace protection and repair in these mice. The TSK/+ mice treated with short-term HGF (low dose and high dose) demonstrated >17% improvement in airspace caliber (Figure 4C, 4D). We also found reduced alveolar oxidative stress by both nitrotyrosine in the HGF-treated mice suggesting that HGF is able to antagonize oxidative stress (Figure 4E, 4F). Using immunoblotting, we analyzed downstream signaling patterns that corresponded to the morphologic and oxidative stress rescue in the TSK/+ mice after HGF augmentation (Figure 4G). We found increased stat3 and akt activation in the TSK/+ lung after HGF augmentation.

### HGF signaling in lung epithelial cells involves conserved activation of downstream cascades

**Figure 5:**
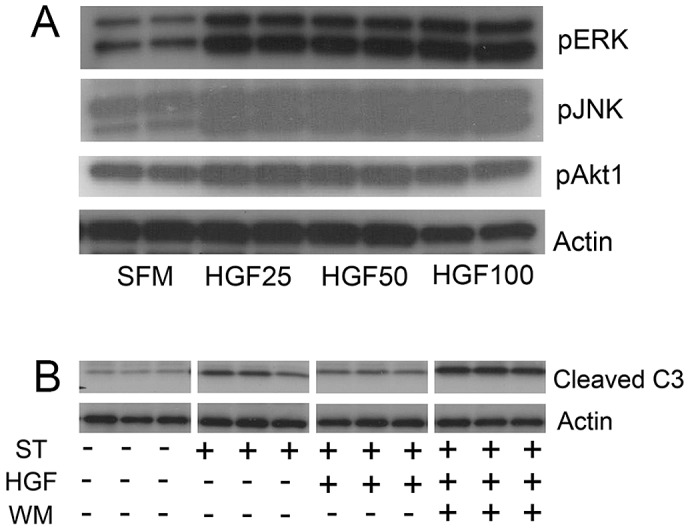
HGF treatment of MLE12 induces prosurvival signaling that protects against alveolar cell death. A. Representative immunoblots of phosphoproteins in mice treated with HGF for 5 min (pERK and pJNK) or 15 min (pAKT) showing dose response. B. Cleaved caspase 3 immunoblotting in MLE12 cells treated with staurosporine with or without HGF or wortmannin. All experiments performed in triplicate.

Since activation of akt and stat3 associated with reparative effects of HGF on pathologic airspace enlargement, we assessed phosphoprotein activation in MLE12 cells treated with HGF. Treatment of MLE12 cells with recombinant human HGF induced activation of ERK, JNK and akt (Figure 5A). Notably, we saw no stat3 activation with HGF treatment (Figure S6A). We assessed whether akt was involved in critical prosurvival events by using staurosporine treatment of MLE12 cells to induce apoptosis. Wortmannin, a known akt inhibitor, inhibited HGF induced akt activation but not ERK activation in MLE12 cells (Figure S6B). We found that staurosporine-induced apoptosis was inhibited by HGF treatment but pretreatment with an akt inhibitor wortmannin fully blocked the protective HGF effect (Figure 5B). Taken together, the in vivo and in vitro findings not only suggest an important role for HGF/c-Met signaling in alveolar epithelial cell survival and maintenance of postnatal airspace homeostasis but also a role for prosurvival signaling cascades as mediators of these events.

## Discussion

Compelling experimental evidence reveals HGF as a “master” driver of organ regeneration and repair processes [20]. HGF promotes functional tissue regeneration in the liver and kidney [21], [22]. Moreover, enhanced HGF secretion accompanies various organ injuries, involving the kidney, liver, lung and heart [23]. Lung injury from bleomycin [24], liver injury from CCl4 administration and renal injury from acid administration [25] in rodent models can be attenuated by exogenous HGF treatment, suggesting that the HGF pathway is involved in conserved organ repair mechanisms [26]. In this study, we showed that alveolar epithelial cell specific HGF signaling is required for airspace homeostasis. We further showed that augmentation of HGF signaling in an established murine model of emphysema can improve airspace enlargement. Finally, a battery of in vivo and in vitro studies utilizing both gain of function and loss of function maneuvers invoke prosurvival phosphoprotein activation and antioxidant signaling as important downstream mediators of the reparative effects of HGF augmentation.

### Requirement of HGF signaling for primary alveolar formation

Our investigation of the loss of function phenotype attached to the cell-specific deletion of *Met* identifies its activation in AECII cells as an important cell-autonomous event in alveolar formation. How does HGF promote alveolarization? The major consequence of *Met* deletion in airspace epithelial cells is the reduced abundance of these cells in the juvenile and adult lungs, increased oxidative stress and inflammation and truncation of the vascular bed. Since whole cell data by our lab and others suggests that c-Met activation induces cell survival and enhanced migration in primary and immortalized alveolar epithelial cells, the septation defect is likely attributable to these mechanisms that converge to compromise septation [8], [27]. Recent work by Factor et al examining mice with hepatocyte- and liver-specific deletion of *Met* similarly demonstrated a profound cell autonomous defect in cell cycle progression, invoking an Erk-1 dependent mechanism [12]. An additional aspect revealed by our study is the truncation of the pulmonary microvasculature which accompanies loss of epithelial expression of c-Met. Yamamoto showed that mice deficient in lung epithelial VEGF-A displayed impaired pulmonary capillary formation and reduced HGF production, implicating the c-Met pathway as a critical mediator of epithelial-endothelial crosstalk in lung homeostasis [10]. Similarly, our findings of combined therapeutic and developmental effects of HGF in the lung support a critical homeostatic role for HGF signaling in the airspace that likely incorporates proliferative, migratory and morphogenic agendas and distinct prosurvival pathways.

### Evidence that genetic and acquired emphysema represent an HGF–deficient state

In the lung, hepatocyte growth factor, secreted by endothelial cells, epithelial cells and interstitial fibroblasts, is sequestered in a precursor state in the extracellular matrix. Although the precise activation events are not well understood in the lung, inflammatory insults trigger the liberation of active HGF and the initiation of HGF/c-Met signaling in many tissues [28], [29]. If HGF signaling is a homeostatic mechanism needed for the maintenance of airspace morphology and cell composition, then an effective deficit in HGF signaling may occur in airspace disorders that are not marked by inflammation. We show that active HGF levels are reduced but c-Met protein levels are preserved in the TSK/+ lung compared with wild-type controls. We suspect this is secondary to defective HGF deposition and activation on the abnormal TSK/+/+ extracellular matrix evident by HGF immunostaining (Figure S2C). Accordingly, downstream HGF signaling is impaired in TSK/+ mice. Since TSK/+ mice exhibit marked postnatal oxidative stress in the lung that promotes airspace enlargement, this lack of maintained or enhanced HGF signaling may be a cause of the enhanced oxidant stress and lead directly to the airspace phenotype [19]. The improved airspace caliber resulting from HGF administration in the TSK/+ mouse suggests suboptimal HGF signaling may be a hospitable context for for airspace protection/repair with HGF administration. As stated above, reduced p-Met activation in the TSK/+ lung combined with enhanced apoptosis and oxidative stress is consistent with impairment in both proximal HGF/c-Met signaling and reparative downstream pathways. Investigators report both reduced and maintained HGF levels in the lungs of patients with COPD/emphysema [30], [31]. Interestingly, patients with acute lung injury typically have increased HGF levels in the bronchoalveolar fluid reflecting a reparative response [32]. Whether those levels are maintained as theinjury evolves is unknown. Infants with bronchopulmonary dysplasia who have reduced HGF levels typically have worse outcomes [33]. Thus, the lack of an *increase* in active HGF signaling in the TSK/+ lung likely reflects an impaired response to epithelial injury.

### Unique reparative effects of HGF augmentation in murine models of emphysema

Evidence that selective cytokines which contribute to alveolar morphogenesis, such as epidermal growth factor (EGF), fibroblast growth factor 10 (FGF10), platelet derived growth factor A (PDGFA), and vascular endothelial growth factor (VEGF), have protective or therapeutic efficacy for animal models of adult airspace disorders is limited [10], [34]–[36]. This limitation largely reflects the extraepithelial effects of these cytokines that may antagonize normal lung repair (reviewed in [37]), a therapeutic requirement for the neonatal rather than adult milieu or simply absence of well-constructed preclinical trials. For example, VEGF is required for airspace formation but overexpression or pharmacologic augmentation of VEGF can have injurious effects, nicely discussed in [38]. Although without a clear role in alveolar formation, keratinocyte growth factor (KGF) is the only growth factor which has a similar functional repertoire as HGF. However, a major limitation for the therapeutic potential of KGF is the probable requirement for pre- or concurrent injury administration (protective rather than therapeutic effects) [3], [39]. In fact, since overexpression of KGF in the murine lung results in severe malformations, the protective/therapeutic window must be carefully defined in neonatal or developing mice [40]. We show that HGF administration has reparative effects in adult TSK/+ mice suggesting a more flexible therapeutic repertoire than KGF. We plan to dissect this difference in future studies.

### Downstream mediators of HGF signaling in alveolar formation or repair

Growth factor induced repair of epithelium may incorporate proliferative, antiapoptotic, migratory and morphogenic agendas [5], [41]. These converge to alter cellular turnover and increase cellular survival, permitting the regeneration of functional structures. We found in the TSK/+ model that stat3 and akt activation, known prosurvival mediators, correlate with the maintenance or reestablishment of airspace integrity.

HGF/c-Met signaling induces stat3 activation frequently resulting in both cellular migration and morphogenesis in a variety of cell systems [42]. Further, a loss of stat3 activation in the murine lung increases susceptibility to hyperoxic injury and overexpression of an activated stat3 confers protection [43], [44]. Similarly, akt is involved in airspace maintenance in a neonatal model of lung injury [45], [46]. HGF-induced akt activation ameliorates cigarette smoke extract induced epithelial cell death [47]. We show here that HGF treatment of immortalized murine lung alveolar epithelial cells (MLE12), an established model of AECII cells, activates akt and appears to mediate prosurvival signaling. We also show that HGF treatment of primary alveolar type II cells promotes survival and expression of antioxidants. Future efforts will focus on dissecting the interface between prosurvival signaling and antioxidant protection in the airspace compartment.

In summary, although alveolar septal loss is the most intractable functional and anatomic lesion in COPD, the molecular basis of this process remains elusive. The mitogenic, motogenic and morphogenic features of HGF make it an attractive candidate mediator of airspace repair. We propose that reduced c-Met activation and expression underlie the inadequate reparative response in the emphysematous lung. Mice deficient in *Met* expression in alveolar epithelial cells display compromised epithelial cell abundance, pruning of the microvascular bed and airspace enlargement. Reduced HGF expression and c-Met activation are evident in inbred mice with genetic emphysema. We have also found that pharmacologic augmentation with recombinant HGF in a murine model of emphysema results in both reduced oxidative stress in the airspace and improved airspace dimension. We define here an important homeostatic role of HGF signaling in airspace formation, maintenance and regeneration suggesting that the HGF/c-Met pathway should be explored for airspace disorders such as bronchopulmonary dysplasia and emphysema.

## Materials and Methods

### Animals

Adult C57Bl6 and TSK/+ mice were housed in a controlled environment and provided with standard water and chow. Animal care was in compliance with IACUC recommendations. Mice conditionally deficient in *Met* expression, *SPC-rtta/+;otet-Cre/+;met^f/f^*, in alveolar epithelial cells were generated by crossing *Met^f/f^* mice harboring an inactivating conditional deletion of exon 16 of the mouse *Met* gene [12] with bitransgenic mice expressing *SPC-rtta;otet-Cre* [11]. Pups resulting from these matings were produced in comparable litter sizes and the genotypes represented in Mendelian ratios. Controls were bitransgenic or single transgenic mice. The mice were housed in a facility accredited by the American Association of Laboratory Animal Care, and the animal studies were reviewed and approved by the institutional animal care and use committee of Johns Hopkins School of Medicine. To induce Cre recombinase, mice were treated with doxycycline (Sigma) at 5 mg/ml in drinking water from conception to time of harvest. Tight-skin (TSK/+) mice backcrossed into a C57Bl/6 background without the pallid allele were generated and maintained as described [19]. Mice were genotyped using standard protocols [11], [12], [48].

### HGF administration to TSK/+ mice

Recombinant HGF provided by Genentech was administered through an intraperitoneal miniosmotic pump placed under isoflurane anesthesia. The pumps were loaded with HGF with carrier in PBS or carrier in PBS alone (control) producing a total daily amount of 25–50 µg for 2 weeks. For intratracheal delivery, HGF was administered per catheter inserted into the tracheal under isoflurane anesthesia and direct inspection.

### Statistical evaluation

Results are expressed as means ± SEM unless otherwise stated. Comparisons between 2 experimental groups were examined using the Student T test or Mann-Whitney rank sum test. Comparisons among 3 or more groups were performed by one-way ANOVA. All statistical analyses were performed with Sigmastat (version 3.5; systat Software, Chicago, IL). A p<0.05 was considered significant.

Additional and more detailed methods are provided in Text S1.

## Supporting Information

Figure S1 HGF and c-Met expression in murine lung. A. HGF staining of 2 week old murine lung with high magnification inset. Scale bar-50 mm. Arrows in inset show HGF staining in lung interstitium. B. Top panel- Representative phase-fluorescent immunohistochemistry of c-Met staining (red) in airway compartment of adult lungs. Note expression in subset of airway epithelial cells (white arrowhead). Blue-DAPI nuclear staining. 20× magnification. N = 4 mice. AW-airway lumen. Bottom panel. Fluorescent immunohistochemistry of c-Met staining in adult lung parenchyma. Note staining of alveolar epithelial cells (arrowhead) and alveolar macrophages (arrow). 20× magnification. N = 3 mice.
(TIF)

Figure S2 Surfactant protein C (SPC) expression is reduced in airspace compartment of 1 month old *SPCMet^f/f^* mice compared with age-matched controls. Top panel-Immunohistochemistry for SPC (red) on sections from control mice (top panel) compared with *SPCMet^f/f^* mice (bottom panel). Right, phase-fluorescent immunohistochemistry of SPC staining. Left, conventional fluorescent immunohistochemistry. 10× magnification. N = 4–6 mice per genotype.
(TIF)

Figure S3 Antioxidant expression in the *Met*-deficient lung. Selective real-time PCR analysis of antioxidant expression in lungs from control and *SPCMet^f/f^* mice.
(TIF)

Figure S4 Matrix deposition in lungs of *Met*-deficient mice. A. Hart's stains of representative lungs of control and *SPCMet^f/f^* mice demonstrate preserved deposition of elastin in the alveoli of mutant mice. 40× magnification. B. Trichrome staining of representative lungs from control and *SPCMet^f/f^* mice shows equivalent deposition of collagen in the bronchovascular compartment. 10× magnification. N = 4–6 mice per genotype.
(TIF)

Figure S5 HGF and c-Met expression in TSK mice. A. ELISA measurement shows preserved expression of c-Met in lungs of 2 wks and 2 month old TSK mice compared with littermate controls. B. Representative immunoblotting of HGFα in 2 wk and 2 month old TSK lung lysates. C. Representative immunohistochemical staining for c-Met (top) and HGF (bottom) in the TSK lung compared with controls showing no overall reduction in c-Met expression in the TSK lung but discontinuous and reduced deposition of HGFα in the TSK lung. D. Alpha smooth muscle actin immunohistochemical staining of lungs of representative wild-type and TSK lung shows minimal fibroblast abundance in alveolar compartment (top panel) but prominent smooth muscle abundance in the airway and vascular walls of both genotypes (bottom panel). N = 4–6 mice per genotype.
(TIF)

Figure S6 The effect of HGF on prosurvival signaling in MLE12 cells. A. Dose response of HGF effect on pstat3 in MLE12 cells. There is no evidence of induction. B. HGF induction of akt1 and ERK1 are inhibited by wortmannin and UO126, respectively, in MLE12 cells. Representative immunoblot of pAKT1 and PERK1 induction after HGF treatment of MLE12 with and without wortmannin or UO126 treatment. WM-Wortmannin, UO1-UO126.
(TIF)

Table S1 Real-time PCR analysis of *Met* and *Hgf* expression in murine wild-type and TSK lung.
(DOC)

Text S1 Supplemental Material and Methods.
(DOC)
