# Supplementary figures and images for: Hepatocyte Growth Factor, a Determinant of Airspace Homeostasis in the Murine Lung
Source: PLoS Genet. 2013 Feb 14;9(2):e1003228. doi: 10.1371/journal.pgen.1003228 (PMC3573081; doi:10.1371/journal.pgen.1003228)

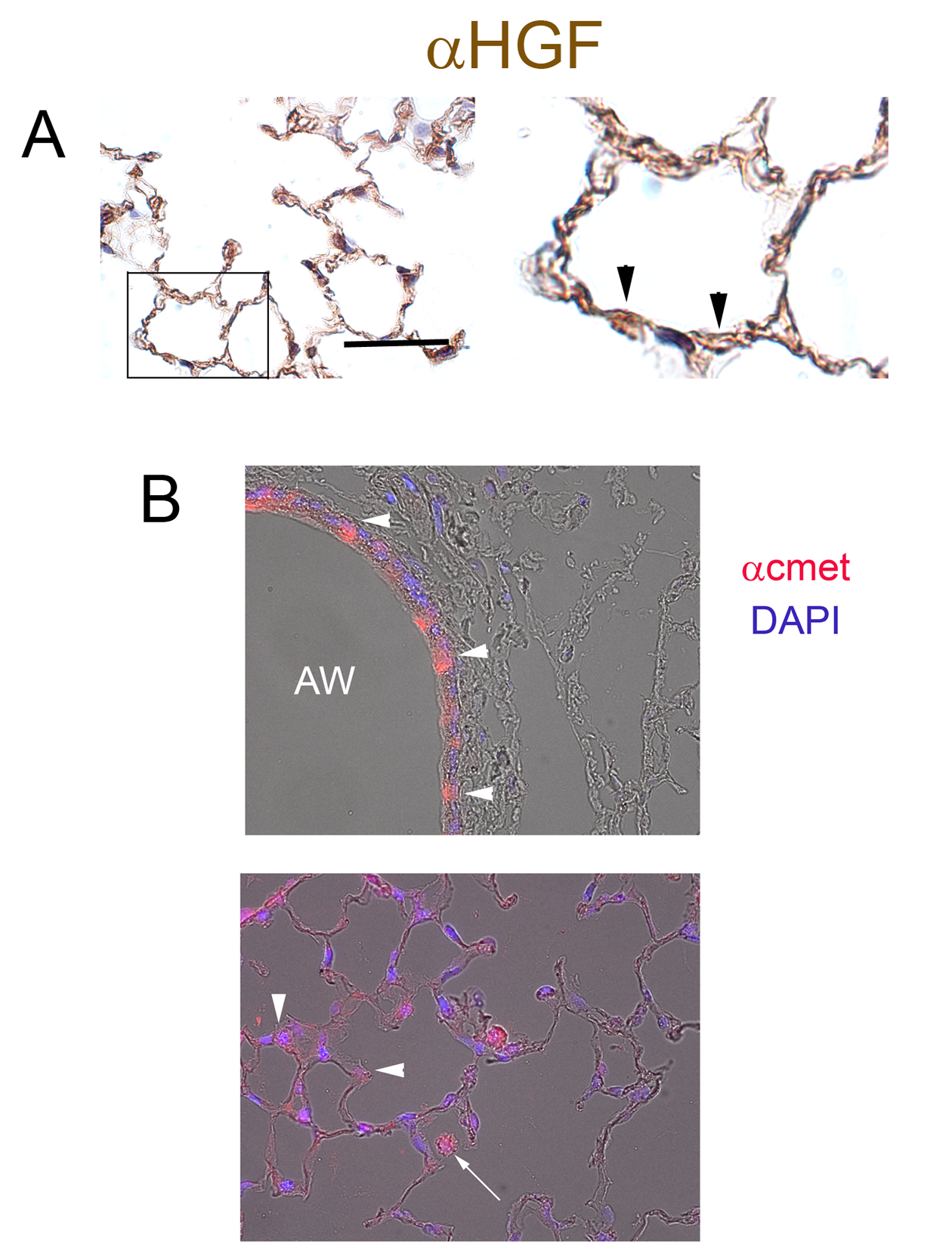

Supplement: Figure S1 — HGF and c-Met expression in murine lung. A. HGF staining of 2 week old murine lung with high magnification inset. Scale bar-50 mm. Arrows in inset show HGF staining in lung interstitium. B. Top panel- Representative phase-fluorescent immunohistochemistry of c-Met staining (red) in airway compartment of adult lungs. Note expression in subset of airway epithelial cells (white arrowhead). Blue-DAPI nuclear staining. 20× magnification. N = 4 mice. AW-airway lumen. Bottom panel. Fluorescent immunohistochemistry of c-Met staining in adult lung parenchyma. Note staining of alveolar epithelial cells (arrowhead) and alveolar macrophages (arrow). 20× magnification. N = 3 mice. (TIF) [file pgen.1003228.s001.tif]

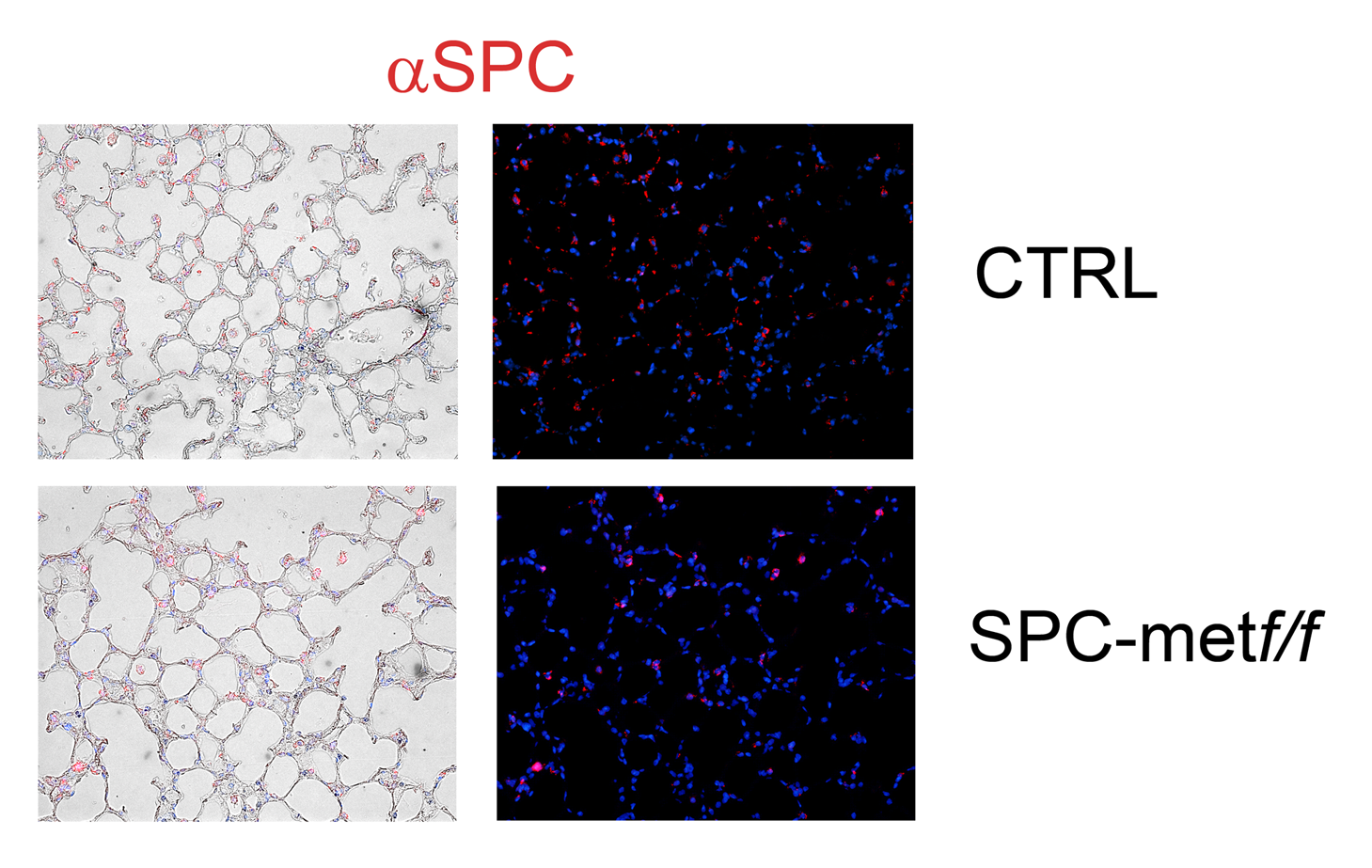

Supplement: Figure S2 — Surfactant protein C (SPC) expression is reduced in airspace compartment of 1 month old SPCMetf/f mice compared with age-matched controls. Top panel-Immunohistochemistry for SPC (red) on sections from control mice (top panel) compared with SPCMetf/f mice (bottom panel). Right, phase-fluorescent immunohistochemistry of SPC staining. Left, conventional fluorescent immunohistochemistry. 10× magnification. N = 4–6 mice per genotype. (TIF) [file pgen.1003228.s002.tif]

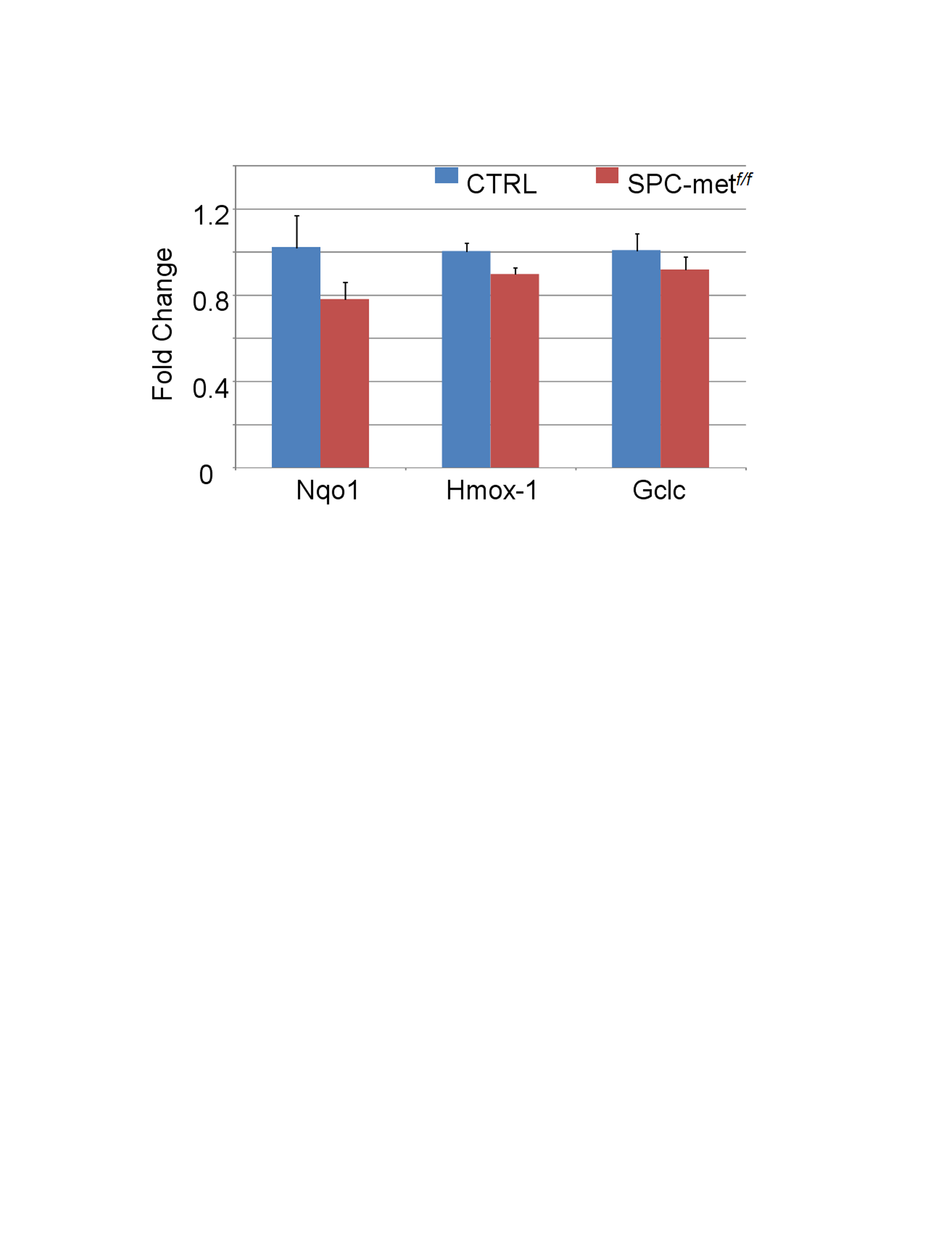

Supplement: Figure S3 — Antioxidant expression in the Met-deficient lung. Selective real-time PCR analysis of antioxidant expression in lungs from control and SPCMetf/f mice. (TIF) [file pgen.1003228.s003.tif]

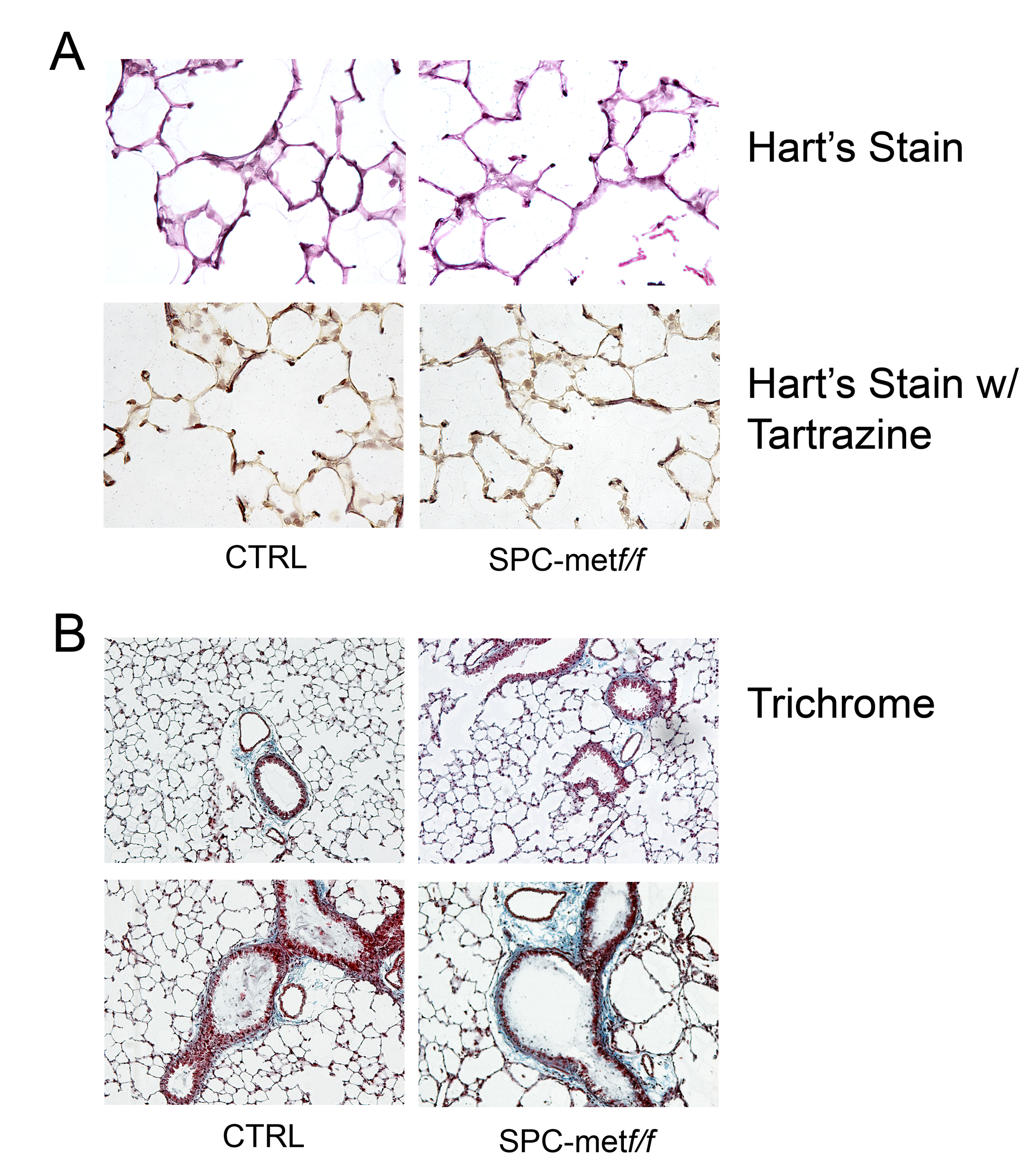

Supplement: Figure S4 — Matrix deposition in lungs of Met-deficient mice. A. Hart's stains of representative lungs of control and SPCMetf/f mice demonstrate preserved deposition of elastin in the alveoli of mutant mice. 40× magnification. B. Trichrome staining of representative lungs from control and SPCMetf/f mice shows equivalent deposition of collagen in the bronchovascular compartment. 10× magnification. N = 4–6 mice per genotype. (TIF) [file pgen.1003228.s004.tif]

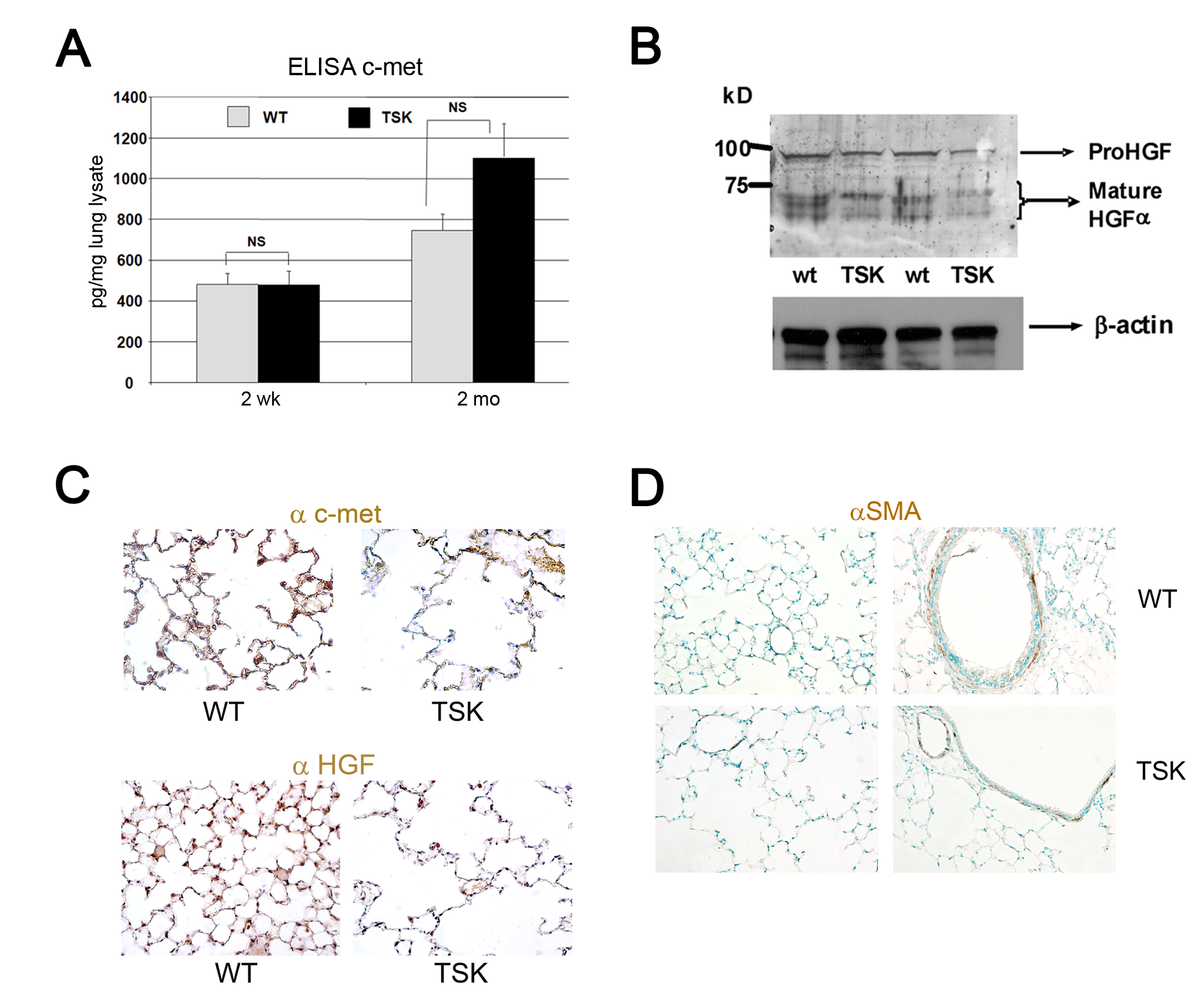

Supplement: Figure S5 — HGF and c-Met expression in TSK mice. A. ELISA measurement shows preserved expression of c-Met in lungs of 2 wks and 2 month old TSK mice compared with littermate controls. B. Representative immunoblotting of HGFα in 2 wk and 2 month old TSK lung lysates. C. Representative immunohistochemical staining for c-Met (top) and HGF (bottom) in the TSK lung compared with controls showing no overall reduction in c-Met expression in the TSK lung but discontinuous and reduced deposition of HGFα in the TSK lung. D. Alpha smooth muscle actin immunohistochemical staining of lungs of representative wild-type and TSK lung shows minimal fibroblast abundance in alveolar compartment (top panel) but prominent smooth muscle abundance in the airway and vascular walls of both genotypes (bottom panel). N = 4–6 mice per genotype. (TIF) [file pgen.1003228.s005.tif]

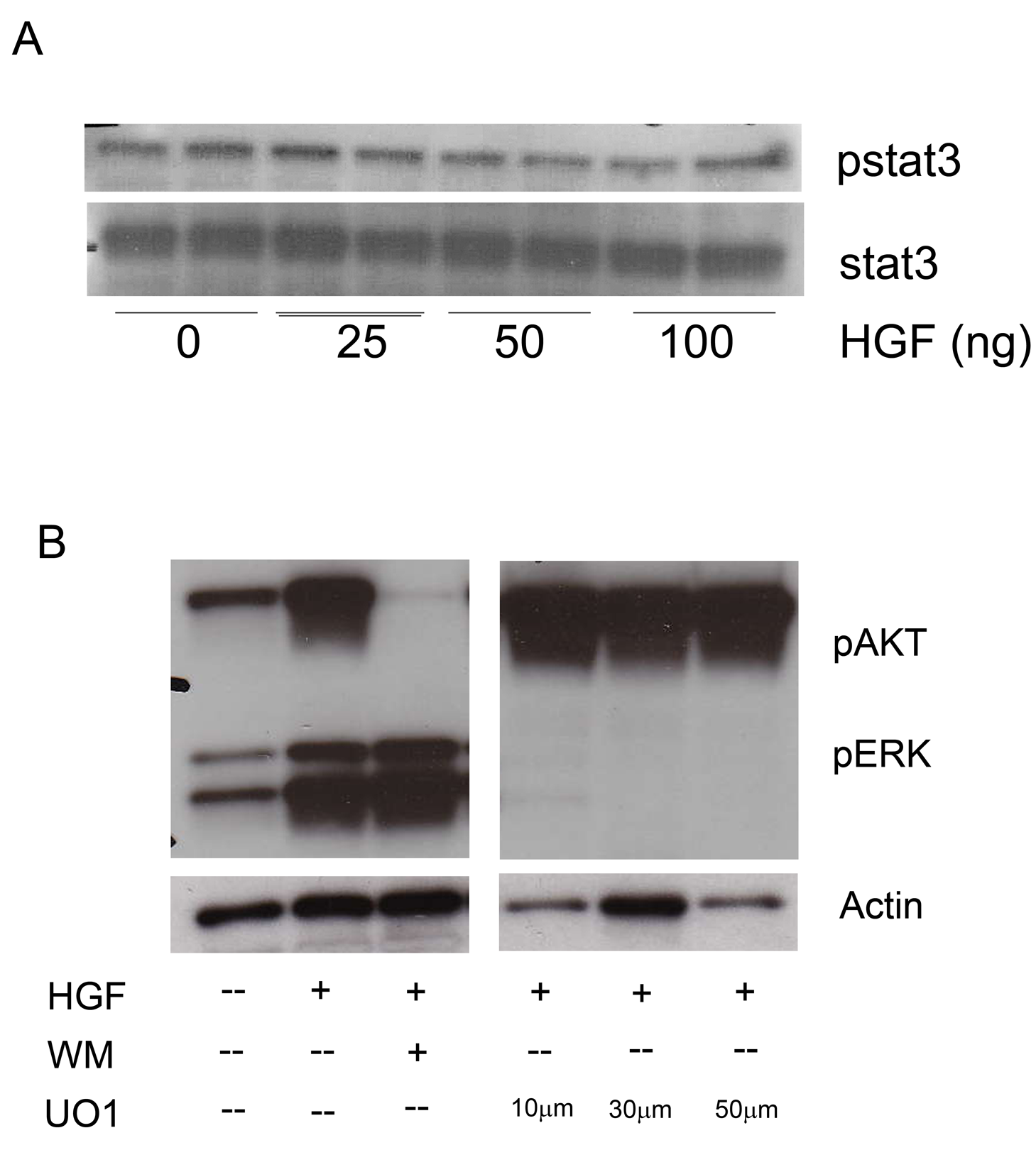

Supplement: Figure S6 — The effect of HGF on prosurvival signaling in MLE12 cells. A. Dose response of HGF effect on pstat3 in MLE12 cells. There is no evidence of induction. B. HGF induction of akt1 and ERK1 are inhibited by wortmannin and UO126, respectively, in MLE12 cells. Representative immunoblot of pAKT1 and PERK1 induction after HGF treatment of MLE12 with and without wortmannin or UO126 treatment. WM-Wortmannin, UO1-UO126. (TIF) [file pgen.1003228.s006.tif]
