# Supplementary material for: Hepatocyte Growth Factor, a Determinant of Airspace Homeostasis in the Murine Lung
Source: PLoS Genet. 2013 Feb 14;9(2):e1003228. doi: 10.1371/journal.pgen.1003228 (PMC3573081; doi:10.1371/journal.pgen.1003228)
Supplement: Table S1 — Real-time PCR analysis of Met and Hgf expression in murine wild-type and TSK lung. (DOC) [file pgen.1003228.s007.doc]

Real Time PCR analysis of c-Met and HGF Expression

in Murine Wild-type and TSK lung

| RNA Source | Transcript | TSK-WT fold change | ttest p-value |
| --- | --- | --- | --- |
| PD14 Lung | *c-met* | 1.28 | 0.12 |
| PD14 Lung | *hgf* | -1.04 | 0.76 |
| 2mo Lung | *c-met* | 1.06 | 0.40 |
| 2mo Lung | *hgf* | 0.94 | 0.79 |
