# Supplementary material for: Hepatocyte Growth Factor, a Determinant of Airspace Homeostasis in the Murine Lung
Source: PLoS Genet. 2013 Feb 14;9(2):e1003228. doi: 10.1371/journal.pgen.1003228 (PMC3573081; doi:10.1371/journal.pgen.1003228)
Supplement: Text S1 — Supplemental Material and Methods. (DOC) [file pgen.1003228.s008.doc]

**Supplementary Materials and Methods**

*Immunoblotting:*  Aliquots of 30 – 50 ug of protein were boiled and then loaded onto Tris-HCL gels and transferred electrophoretically to nitrocellulose membranes. Membranes were incubated with the primary antibody for 1 h at room temperature. Detection was by the Pierce West Dura ECL detection system. Primary antibodies and dilutions were as follows: b-actin (Abcam), HGFa (Sigma), pAKT/AKT (Cell Signaling), pERK/ERK (Cell Signaling), pJNK/JNK (Cell Signaling), pstat3/stat3 (Cell Signaling), active and total caspase 3 (Santa Cruz). Densitometry was performed using ImageJ.

*Isolation of murine ATII cells:* Isolation of mouse alveolar type 2 epithelial cells was performed using previously described protocols with modification(Corti et al. 1996; Beck et al. 2009; Mercer et al. 2009). Briefly, lungs from 6-8 wk old mice were perfused with PBS, and filled with 2 ml of 20U/ml of dispase II ( Roche,Indianapolis, IN). Lungs were then removed to 6 ml dispase (4U/ml) / DNAse II (40U/ml Roche, Indianapolis, IN) in 50 ml tube and incubated for 30 min at 37C, followed by mincing and sequentially passing thru cell strainers(BioExpress, China). The suspension was centrifuged at 900rpm for 8 min at 4C and resuspended in 6 ml of culture media. For hematopoietic cell removal, cells were plated on antibody coated Petri dishes ( CD 16/32, CD 45, CD 90 and TER 119) and incubated at 37C for 35min – 1 h. Floating cells were collected , centrifuged , resuspended in culture media and plated overnight for fibroblast removal and cells recovery. ATII expression was validated by cytospin analysis and SPC staining and confirmed >80% purity.

*Immunohistochemistry:* Tissue sections were deparaffinized, rehydrated in an ethanol series and subjected to epitope retrieval using standard maneuvers. Sections were blocked for non-specific binding with 3% normal serum from chicken and incubated with the primary antibodies for 1 hour at room temperature. For DAB immunohistochemistry, following incubation with the primary antibody overnight at 4°C, slides were washed with PBST, incubated with an appropriate biotinylated secondary antibody (Jackson ImmunoResearch) and developed by using ABC and DAB detection reagents (Vector Laboratories). Sections were counterstained with either hematoxylin or methyl green per standard protocol. For immunofluorescence, sections were then incubated with secondary antibodies at 1:200 for 30 minutes at room temperature (Molecular Probes). Sections were counterstained with 4’,6’-diamidinio-2-phenylindole (DAPI) and mounted with Vectashield hard set mounting medium (Vector LabsAntibodies were used at the following concentrations: HGF 1:50 (Sigma), cmet 1:100 (Santa Cruz), pmet 1:100 (Cell Signaling), Nitrotyrosine 1:250 (Dako), Surfactant protein C 1:100 (Chemicon), Mac3 1:50 (Serotec), and Ki67 1:100 (Dako). Quantitative immunohistochemistry was performed by normalizing staining to either total cell count or tissue area as indicated using NIS or Metamorph software.

*Immunofluorescent staining to detect the endothelial cells on mouse lung paraffin-embedded sections:* After deparaffinization and hydration, we steamed the control and knock down-tissue sections in Borg Decloaker (Biocare Medical, Concord, CA) for 10 min for antigen retrieval as previously described (Le et al. 2008; Le et al. 2009). Endothelial cells were detected by polyclonal goat anti-thrombomodulin (R&D Systems; diluted 1:2,000) which was diluted in antibody diluent purchased from DakoCytomation. The secondary antibody Alexa Fluor 488 donkey anti-goat was purchased from Invitrogen, Carlsbad, CA. We used 4_,6-Diamidino-2-phenylindole (Invitrogen) to stain cellular nuclei. Staining control sections were incubated with similar concentrations of isotype-specific (IgG) inteads of primary antibody, followed by the same secondary antibodies. Four random fields of each slide were analyzed under Zeiss LSM 510 META confocal microscope at 40X magnification (Ross Confocal Microscope facility).

*Morphometry:* For histologic and morphometric analyses, mouse lungs were inflated at 25cm H20 and fixed with 4% paraformaldehyde (PFA) in low melt agarose. The lungs were equilibrated in cold 4% PFA overnight, sectioned and then embedded in paraffin wax. Sections were cut at 5 µm and either stained with hematoxylin and eosin (H&E) or processed for immunohistochemistry. Morphometric measurements were performed on H&E stained sections taken at intervals throughout both lungs. Slides were coded, captured by an observer, and masked for identity for the groups. Ten to fifteen images per slide were acquired at 20x magnification and transferred to a computer screen. Mean chord lengths and mean linear intercepts were assessed by automated morphometry with a macro operation performed by Metamorph Imaging Software (Universal Imaging, Molecular Devices, Downingtown, PA).

*ELISA Assays:* HGF levels by ELISA (R&D Systems) were measured in serum and bronchoalveolar lavage samples from mice treated with recombinant human HGF pump 50 ug/day for 3 days or PBS pump as per protocol. Murine HGF levels in TSK mice and wild type controls were measured using the HGF ELISA kit standard protocol, (Institute of Immunology, Tokyo, Japan). Murine cmet levels were measured in lung lysates using commercial assay (R&D Systems).

*Whole Cell Treatment Protocols:* MLE12 cells were serum-starved for 24h and then treated with recombinant h-HGF for defined time points and lysates harvested for phosphoprotein analysis. Wortmannin was administered at designated doses 1 hour before HGF treatment for the akt inhibition experiments. Staurosporine was added 3 hours after the HGF treatment. For survival studies, freshly isolated ATII cells were treated with recombinant h-HGF for designated time periods in serum free medium and then assessed for cell survival with hemacytometer and trypan blue exclusion. For RT-PCR, the cells were harvested after 2h incubation with h-HGF or vehicle.

Real-time PCR: Total RNA isolated from cells with Trizol-based protocol was treated with DNase and reverse-transcribed as described (Calvi et al.). The expression levels of target genes were determined in triplicate or quadruplicate from the standard curve and normalized to Gapdh mRNA level.

*Cell Proliferation Assay.* The MLE12 cell line, an immortalized mouse lung epithelial cell line that maintains characteristics of type II alveolar epithelial cells(Yei et al. 1994), was a kind gift from Dr. Jeffrey Whitsett (Univ. of Cincinnati) and was maintained in Dulbecco’s minimal essential medium:F12, 1:1, (DMEM/F12) media supplemented with 2% FCS. For proliferation assays, serum-starved cells were incubated for the designated time periods after treatment or transfection and then assayed per protocol with the Promega CellTitre 96 Nonradioactive Proliferation Assay.

*Cell Scattering Assay.* Protocol modified from (Santos et al. 1993)**.** Briefly, MLE12 cells were seeded at low density in a six well plate. After serum starvation for 4 hours in 0.05% FBS, cells were stimulated with or without 10ng of HGF/ml for 24h. The morphology of the paraformaldehyde-fixed cells were photographed under phase-contrast microscopy.

**References:**

Beck JM, Preston AM, Wilcoxen SE, Morris SB, Sturrock A et al. (2009) Critical roles of inflammation and apoptosis in improved survival in a model of hyperoxia-induced acute lung injury in Pneumocystis murina-infected mice. Infect Immun 77(3): 1053-1060.

Calvi CL, Podowski M, D'Alessio FR, Metzger SL, Misono K et al. Critical transition in tissue homeostasis accompanies murine lung senescence. PLoS One 6(6): e20712.

Corti M, Brody AR, Harrison JH (1996) Isolation and primary culture of murine alveolar type II cells. Am J Respir Cell Mol Biol 14(4): 309-315.

Le A, Zielinski R, He C, Crow MT, Biswal S et al. (2009) Pulmonary epithelial neuropilin-1 deletion enhances development of cigarette smoke-induced emphysema. Am J Respir Crit Care Med 180(5): 396-406.

Le A, Damico R, Damarla M, Boueiz A, Pae HH et al. (2008) Alveolar cell apoptosis is dependent on p38 MAP kinase-mediated activation of xanthine oxidoreductase in ventilator-induced lung injury. J Appl Physiol 105(4): 1282-1290.

Mercer PF, Johns RH, Scotton CJ, Krupiczojc MA, Konigshoff M et al. (2009) Pulmonary epithelium is a prominent source of proteinase-activated receptor-1-inducible CCL2 in pulmonary fibrosis. Am J Respir Crit Care Med 179(5): 414-425.

Santos OF, Moura LA, Rosen EM, Nigam SK (1993) Modulation of HGF-induced tubulogenesis and branching by multiple phosphorylation mechanisms. Dev Biol 159(2): 535-548.

Yei S, Bachurski CJ, Weaver TE, Wert SE, Trapnell BC et al. (1994) Adenoviral-mediated gene transfer of human surfactant protein B to respiratory epithelial cells. Am J Respir Cell Mol Biol 11(3): 329-336.
